# Leftover Medicine: A Perspective and Initiative for Environmental Sustainability in Japanese Dialysis Facilities

**DOI:** 10.31662/jmaj.2024-0436

**Published:** 2025-11-14

**Authors:** Kei Nagai, Tsutomu Kuno, Nanae Matsuo, Yutaka Koda, Kanji Shishido

**Affiliations:** 1 Department of Nephrology, Hitachi General Hospital, Hitachi, Japan; 2 Department of Nephrology, Faculty of Medicine, University of Tsukuba, Tsukuba, Japan; 3 Ikebukuro Kuno Clinic, Tokyo, Japan; 4 Division of Nephrology and Hypertension, Department of Internal Medicine, The Jikei University School of Medicine, Tokyo, Japan; 5 Koda Medical and Dialysis Clinic, Niigata, Japan; 6 Kawasaki Clinic, Kawasaki, Japan

**Keywords:** chronic kidney disease, hemodialysis, leftover medicine, sustainability

## Short Communication

Health care undoubtedly affects the environment. Greenhouse gas emissions from medical care in Japan account for 5%-6% of all industrial emissions ^(1)^. Dialysis therapy for end-stage kidney disease also requires substantial amounts of medical materials and resources. Among various medical practices, kidney health care has a particularly high environmental impact, including disproportionate contributions to carbon emissions and climate change ^(2)^. “Green nephrology” has recently been developed to raise awareness among medical personnel and provide sustainable treatment with minimal impacts on the environment ^(2)^. Surprisingly, approximately half of carbon emissions from dialysis therapy are due to the procurement of pharmaceuticals, exceeding the contributions of medical materials and resources ^(3)^. Minimizing drug losses would thus represent an effective and feasible method of reducing carbon emissions. A certain number of drugs might be disposed of for various reasons after prescription, but actual initiatives regarding leftover medicine are unclear. After the first “Green Survey” conducted in Japan in 2023 to evaluate environmental awareness among dialysis providers ^(4)^, we conducted another nationwide survey of dialysis facilities in September 2024, with the theme of leftover medication. Questionnaires were created in Google Forms and sent to all members of the Japanese Association of Dialysis Physicians who provide hemodialysis and/or peritoneal dialysis treatment, including both hospital and clinic physicians, representing 885 dialysis facilities. Responses were obtained from 219 members in 45 of 47 prefectures, and all responses were valid.

**Figure 1.**
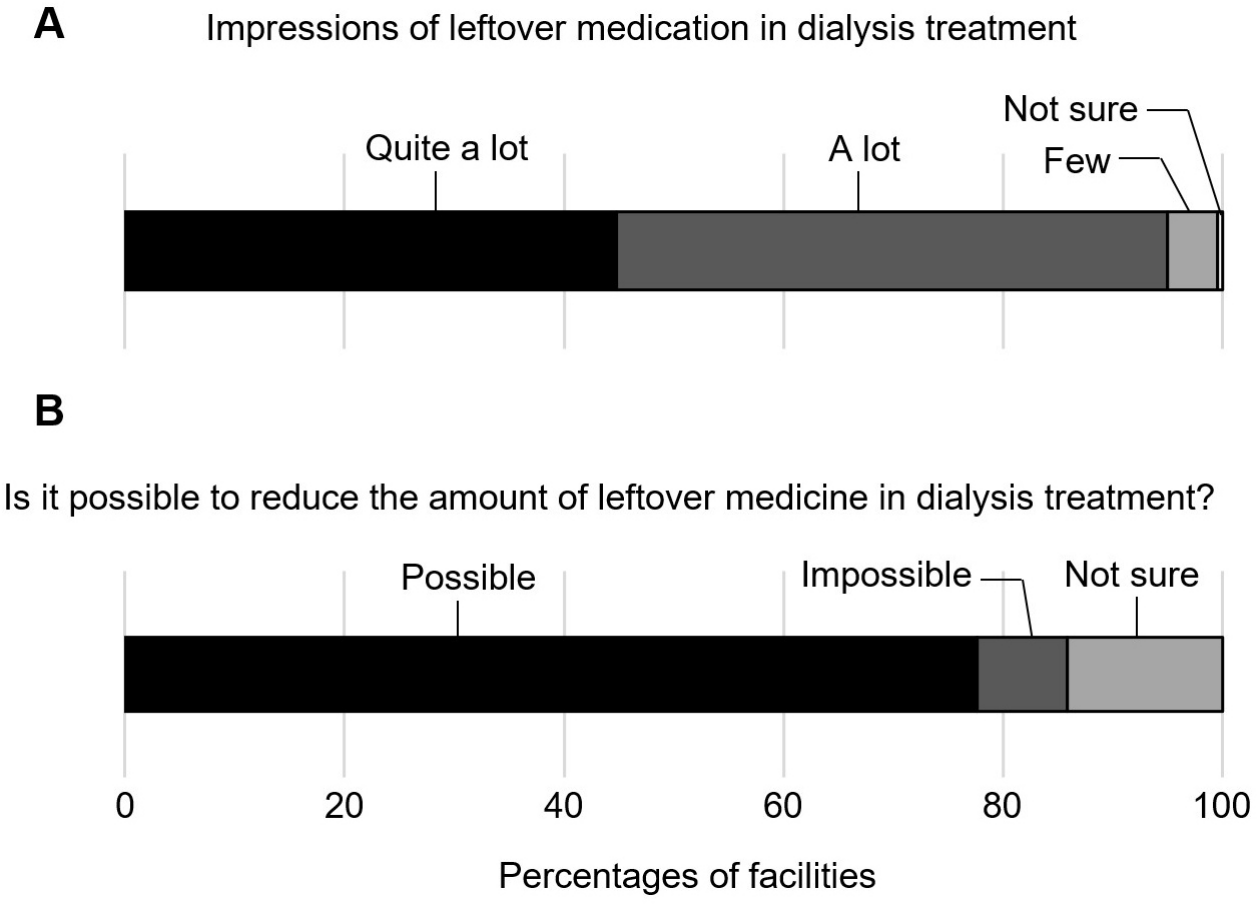
Providers’ impressions regarding leftover medication in dialysis therapy. Between August and September 2024, an online nationwide survey regarding leftover medication was sent to members of the Japanese Association of Dialysis Physicians, working across 885 dialysis facilities in Japan. Responses were received from 219 members, all of which were valid. Answers for questions regarding amounts of leftover medicine (A), and the prospects for reducing amounts of leftover medicine (B) are indicated.

**Figure 2.**
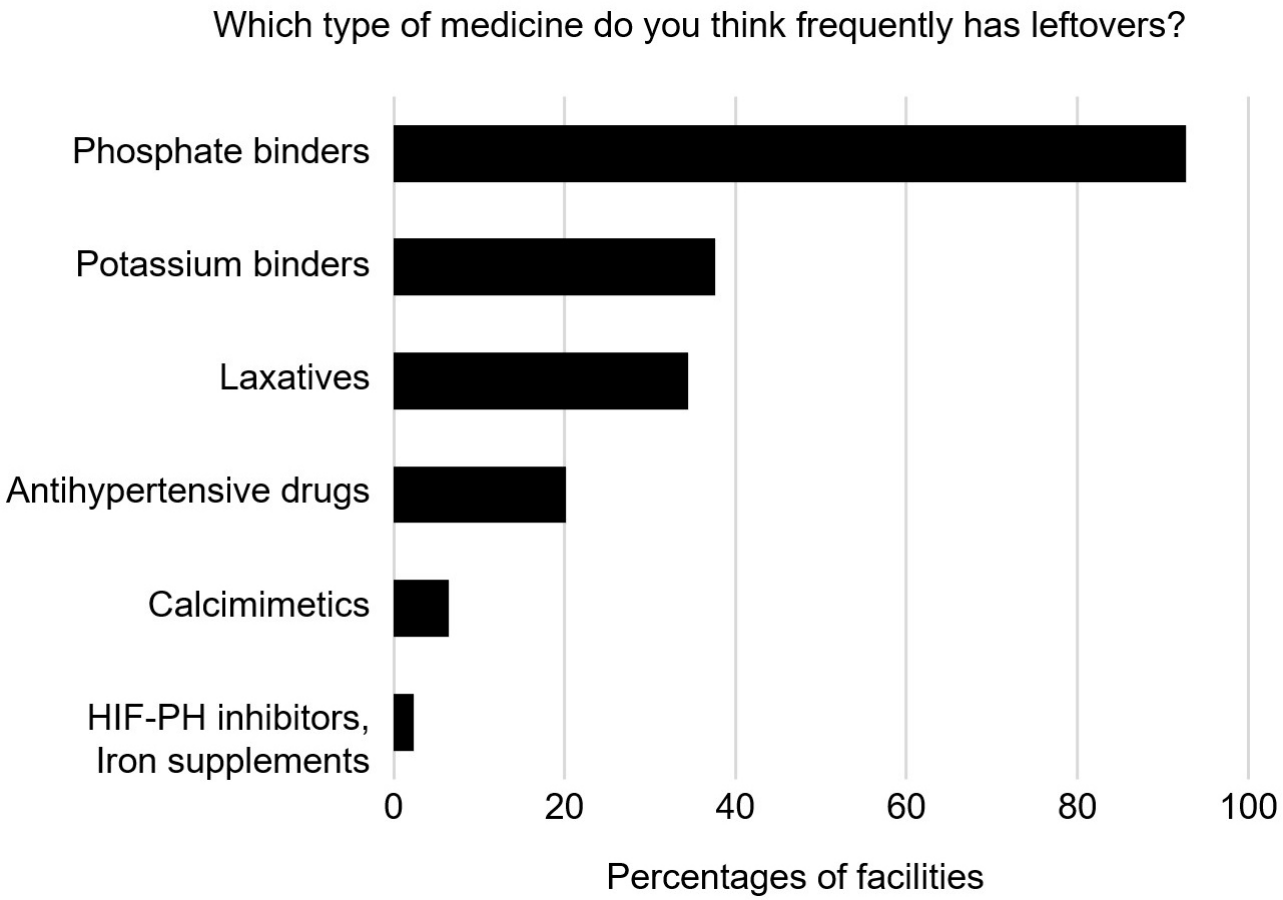
Providers’ views on drug type and leftover medication in dialysis therapy. Answers to questions about the types of drugs likely to be leftover are shown. HIF-PH: hypoxia-inducible factor prolyl hydroxylase.

Most respondents answered that the amount of leftover medicine was either “quite a lot” (44.7%) or “a lot” (50.2%), indicating relatively high awareness of the problem (Figure 1A). Furthermore, approximately 80% of dialysis providers considered that amounts of leftover medicine could be reduced in the future (Figure 1B). The drugs that medical providers at dialysis facilities deemed to be by far the most prone to causing inappropriate patient use were phosphate binders (92.7%), followed by potassium binders (37.6%), laxatives (34.4%), and antihypertensive drugs (20.2%) (Figure 2). This seems reasonable, given that Ohya et al. ^(5)^ previously reported results from another survey on medications in 524 patients on maintenance hemodialysis. That survey revealed that adherence was worst for phosphate binders, with 23.3% of respondents answering that they did not use these agents as instructed. Similarly, Iwashita et al. ^(6)^ showed that phosphate binders composed a high proportion of the tablets taken (7 of the average total of 16.4 tablets per day). Taken together, our survey on the views of providers regarding leftover medicine for patients on dialysis from the perspective of environmental sustainability revealed positive results, showing strong awareness and potential for solutions regarding the remaining amounts of drugs represented by phosphate binders as drugs with many tablets or doses.

We further asked an open-ended question on the current situation regarding efforts to tackle the issue of leftover medicine. Table 1 shows current initiatives by dialysis therapy providers to reduce leftover medicine. Among the 219 facilities, and allowing duplicate answers, the most common measure was to make single-dose packaging for each time medications had to be taken (112 facilities, 51.1%), followed by reducing the frequency of taking medicines (39 facilities, 17.8%), and using combination drugs. Regarding regular prescriptions issued on a weekly or monthly basis, doctors adjust the number of days on the prescription based on the amount of leftover medicine for the patients (13 facilities, 5.9%), encouraging physicians to review regular prescriptions (12 facilities, 5.5%), and collecting leftover medicine several times a year for organization in dialysis facilities (5 facilities, 2.3%).

**Table 1.** Current Initiatives for Reducing Leftover Medicine by Dialysis Therapy Providers.

Category	Initiatives	Number of facilities	Percentages
Prescription details (dosage, form, number)	Single-dose packaging	112	51.1%
	Reduced frequency of taking medication	39	17.8%
	Combination drugs	20	9.1%
	Reduction in number of drugs/tablets	14	6.4%
	Switching from oral to injectable drugs	6	2.7%
	Consideration of dosage form (e.g., chewable)	2	0.9%
	Reduce oral drugs by high-efficiency dialysis	1	0.5%
Regular prescriptions	Adjusting the number of days on the prescription on the basis of the amount of leftover medicine for the patients	13	5.9%
	Periodic prescription review	12	5.5%
	Shorten interval for regular prescriptions	5	2.3%
	Regular collection of leftover medicine (several times a year)	5	2.3%
	Taking medication during dialysis session/dialysis room management of medication	2	0.9%
Patient education	Health care providers and patients talk to each other regarding leftover medicine and health problems from poor medication adherence	19	8.7%
Cooperative system	Family/caregiver	8	3.7%
	Visiting pharmacists and nurses	8	3.7%
	Checking and organizing leftover medicine at local pharmacy	7	3.2%
Devising ways to improve medication habits	Medicine case, calendar	14	6.4%
	Leftover medicine (brown) bag	7	3.2%
	Use of information technology	2	0.9%

Although initiatives led by health care providers are certainly important, patient education is also indispensable. Nineteen facilities (8.7%) mentioned the need for health care providers and patients to talk to each other about reasons for leftover medicines and the health problems that can arise from poor compliance with medication prescriptions. Some facilities also mentioned the importance of building a cooperative system with family members, caregivers, visiting pharmacists, visiting nurses, and involvement of local pharmacies for checking and organizing leftover medicine. Twenty-one facilities (9.6%) responded that they had measures in place to encourage good habits in taking medication, such as medicine cases, medicine calendars, and management using leftover medicine bags (also called the “Brown Bag Movement,” as described below).

Generally, the greenhouse gas emissions of pharmaceuticals derive not from direct emissions from factories but rather from the so-called “Scope 3 emissions,” which account for more than 80% of the total, and include the procurement of raw materials, the production and importation of active pharmaceutical ingredients, transportation, and disposal processes ^(7)^. The environmental impact of drugs provided to patients on dialysis is far greater than might initially be imagined. Drug loss, when a necessary medication is not actually taken, should therefore be avoided both from an economic standpoint and for the global environment.

Maintaining medication adherence using single-dose packages or combination tablets, and minimizing the number of times a medication must be taken by switching to long-acting drugs (e.g., for antihypertensive drugs) are considered reasonable options for reducing the risk of missing medication. In addition, rationalization for reducing numbers of tablets should also be tried, considering effectiveness and side effects. For instance, with phosphate binders often taken with laxative, the number of laxative tablets may be able to be reduced by switching to serum phosphorus-lowering drugs such as tenapanor, which tends to make soft stools.

Although phosphate binders and potassium binders are theoretically unnecessary if the dialysis is sufficiently efficient to match the amount ingested, a combination of excessive phosphorus and potassium intake by the patient and time constraints on dialysis make oral binders necessary in common dialysis practice. Conversely, as an opinion expressed in this survey (Table 1, 1 facility, 0.5%), reducing the use of phosphate and potassium binders by extending dialysis time to improve dialysis efficiency is performed daily in some dialysis facilities. The effects of extending dialysis time on patient quality of life and the working conditions of health care providers remain contentious, as does the balance of effects on greenhouse gas emissions between the impact of dialysis fluid and electricity with prolonged dialysis and mitigation through reduced medication. Switching from oral to injectable drugs to reduce the number of medications taken may be effective in terms of preventing drug loss. However, differences in environmental impact should be carefully considered when comparing oral and injectable drugs. Injectable drugs require high levels of sterilization, in addition to refrigeration, metal needles, and other equipment that have been deemed to involve higher greenhouse gas emissions than do oral drugs ^(8)^.

The Brown Bag Movement is an activity known among progressive pharmacists. This pharmacy service began in the United States in the 1980s, involving patients bringing their daily medications in a brown paper bag to the pharmacy, allowing pharmacists to check for any problems with drug interactions or medication adherence. In Japan, from 2010, pharmacists in several regions started cooperating with medical associations and pharmacists’ associations to implement “drug savings” with the aim of reducing drug costs ^(9)^. Although no reports have described the Brown Bag Movement being organized for patients on dialysis, the Japanese Association of Dialysis Physicians envisages working with pharmacists to reduce the amounts of leftover medicine in cooperation with local pharmacists’ associations and medical associations.

In summary, this survey highlighted the issue of leftover medicine, which has become a topic attracting attention among dialysis providers in Japan. Now is the right time to take action toward sustainable kidney health care.

## Article Information

### Acknowledgments

We thank the Japanese Association of Dialysis Physicians (JADP) and its president, Dr. Tadao Akizawa, for their cooperation with this survey.

### Author Contributions

Conceptualization, Investigation, and Writing - Original Draft Preparation: Kei Nagai and Tsutomu Kuno. Data collection: Tsutomu Kuno, Yutaka Koda, and Kanji Shihido. Supervision and Writing - Review and Editing: Nanae Matsuo, Yutaka Koda, and Kanji Shihido. All authors agree with the manuscript results, conclusions, and decision to publish.

### Conflicts of Interest

Kei Nagai received lecture fees from AstraZeneca K.K., Nippon Boehringer Ingelheim Co., Ltd., Mochida Pharmaceutical Co., Ltd., Kyowa Kirin Co., Ltd., Kissei Pharmaceutical Co., Ltd., Torii Pharmaceutical Co., Ltd., Fuso Pharmaceutical Industries, Ltd., Astellas Pharma Inc., and Daiichi Sankyo Healthcare Co., Ltd. Tsutomu Kuno received lecture fees from Kyowa Kirin Co., Ltd. and Nipro Co. Nanae Matsuo received lecture fees from Baxter International Inc. and Terumo Co., Ltd..

Yutaka Koda received lecture fees from Fuso Pharmaceutical Industries, Ltd., Nikkiso Co., Ltd., and JMS Co., Ltd. Kanji Shishido received lecture fees from Kyowa Kirin Co., Ltd., Astellas Pharma Inc., Fuso Pharmaceutical Industries, Ltd., AstraZeneca K.K., Kissei Pharmaceutical Co., Ltd., Sanwa Kagaku Kenkyusho Co., Ltd, Ono Pharmaceutical Co., Ltd., Kaneka Co., and JMS Co., Ltd.

### IRB Approval and Consent

Because this study did not involve human participants as patients or animals, ethics approval was not required.

### Data Availability

The data that support the findings of this study are available on reasonable request from the corresponding author (Kei Nagai).
